# The Protective Effect of Ursolic Acid on Unilateral Ureteral Obstruction in Rats by Activating the Nrf2/HO-1 Antioxidant Signaling Pathway

**DOI:** 10.1155/2022/3690524

**Published:** 2022-08-25

**Authors:** Jun Pei, Moudong Wu, Shuyu Cai, Jinpu Peng, Xiong Zhan, Dan Wang, Wei Wang, Nini An

**Affiliations:** ^1^Department of Pediatric Surgery, Guizhou Provincial People's Hospital, Guiyang 550002, China; ^2^Department of Nephrology, First Affiliated Hospital of Guangxi Medical University, Nanning 530021, China

## Abstract

Renal interstitial fibrosis is a common pathological feature of a variety of kidney diseases that progress to end-stage renal disease. The excessive deposition of extracellular matrix (ECM) is a typical pathological change of renal interstitial fibrosis. The production of reactive oxygen species in renal tubules is an important factor leading to the development of renal interstitial fibrosis. Ursolic acid (UA) is a natural pentacyclic triterpene carboxylic acid compound widely found in plants. It has anti-inflammatory, antioxidant, and antitumor cell proliferation effects. It can reduce the development of fibrosis by inhibiting the oxidative stress response of the liver; there is currently no relevant research on whether UA can protect the renal interstitial fibrosis by resisting oxidative stress in the kidneys. In this study, our purpose is to investigate the effect of ursolic acid on renal interstitial fibrosis after unilateral ureteral obstruction (UUO) in rats and its related mechanisms. We established a UUO model by surgically ligating the right ureter of the rat and instilling UA preparation (40 mg/kg/d) through the stomach after the operation, once a day for 7 days. We found that UUO caused impaired renal function, increased pathological damage, increased renal interstitial fibrosis, increased apoptosis, increased oxidative stress damage, and decreased antioxidants. However, after UA preparations were given, the abovementioned damage was significantly improved. At the same time, we also found that UA preparations can significantly increase the relative expression of Nrf2/HO-1 signaling pathway in kidney tissue after UUO. In order to further verify whether the Nrf2/HO-1 signaling pathway is involved in the development of renal interstitial fibrosis, we injected zinc protoporphyrin (ZnPP, 45 umol/kg), a specific blocker of the Nrf2/HO-1 signaling pathway, into the intraperitoneal cavity after UUO in rats and before the gastric perfusion of ursolic acid preparations. Subsequently, we observed that the protective effect of UA on renal interstitial fibrosis after UUO in rats was reversed. Combining all the research results, we proved that UA has a protective effect on renal interstitial fibrosis after UUO in rats, which may be achieved by activating the Nrf2/HO-1 signaling pathway.

## 1. Introduction

Renal interstitial fibrosis is a common pathological feature of many chronic kidney diseases, and it is also considered to be a common process of progression to end-stage renal disease. The excessive accumulation of extracellular matrix (ECM) is considered to be a typical pathological change in the development of renal interstitial fibrosis [1]. In previous studies, it has been confirmed that the enhancement of oxidative stress or the imbalance of the redox reaction is closely related to renal function damage [2]. A large number of studies have proved that oxidative stress is an important factor leading to tubular interstitial fibrosis, and the oxygen free radicals produced in the process can accelerate the fibrosis process mediated by transforming growth factor-*β*1 (TGF-*β*1) [2].

Nuclear factor E2-related factor 2 (Nrf2) is a nuclear transcription factor with a basic leucine structure and an important central regulator of the body's cell antioxidative stress response. Under normal circumstances, Nrf2 binds to its inhibitor Kelch-like epichlorohydrin-related protein-1 (Keap1) and is widely distributed in the cytoplasm in an inactive form [3–5]. When the body is stimulated by the oxidative stress response, the conformation of Keap1 changes and Nrf2 is released into the nucleus. And through the combination with antioxidant genes on the antioxidant response element (ARE), it induces the production of downstream antioxidants, for example, heme oxidase-1 (HO-1), superoxide dismutase (SOD), *γ*-glutamylcysteine synthase (*γ*-GCS), and quinone oxidoreductase-1 (NQO1), so as to play a defensive role on the cell. Studies have suggested that Nrf2 not only has the effect of antioxidative stress, but also can attenuate the production of extracellular matrix (ECM) mediated by TGF-*β*1. Therefore, activating the production of Nrf2 may have a potential therapeutic effect on renal interstitial fibrosis.

Ursolic acid (UA) is a low-toxic pentacyclic triterpene carboxylic acid compound, which has anti-inflammatory and antioxidant activities, inhibiting tumor cell proliferation and protecting liver damage. It is widely found in many plants. Some scholars have proposed that UA can inhibit liver oxidative stress and liver fibrosis by activating NOX4/ROS and RhoA/ROCK1 signaling pathways [6]. At the same time, UA can also play a protective role against renal ischemia-reperfusion injury by activating the STAT3/NF*κ*B signaling pathway [7]. However, whether the application of UA also has a protective effect on renal interstitial fibrosis and the related mechanisms are still unclear. In this study, we used UA to study the renal interstitial fibrosis model after UUO in rats to determine whether UA has a protective effect on renal interstitial fibrosis and its related mechanisms. It has good application prospects in the treatment of clinical renal interstitial fibrosis.

## 2. Materials and Methods

### 2.1. Animal

Twenty-four adult male Sprague-Dawley rats (12 weeks, weighing 250–300 g) (license number: SCXK (Liao) 2019–0024), purchased from Liaoning Changsheng Biotechnology Co., Ltd. They were housed at temperatures ranging from 23 to 25°C under 12-h light/12-h dark cycles. The rats had free access to food and water. This experiment was approved by the Ethics Committee of the Guizhou University of Traditional Chinese Medicine (20190012). This experiment was performed in accordance with relevant guidelines and regulations. This study is reported in accordance with ARRIVE guidelines.

### 2.2. Animal Grouping and Establishment of Unilateral Ureteral Obstruction Model

*E*, after the right ureteral obstruction, before the stomach was perfused with ursolic acid preparations, intraperitoneal injection of zinc protoporphyriTwenty-four adult male SD rats were randomly assigned to 4 groups, 6 in each group, namely, the sham operation group (group S), the control group (group C), the experimental group (group E), and group ZnPP + *E* after intraperitoneal injection of zinc protoporphyrin (ZnPP), a specific blocker of Nrf2 signaling pathway. Rats in each group were anesthetized by the intraperitoneal injection of 5% pentobarbital sodium (5 mg/kg); group S rats only bluntly separated the right ureter, but not ligated; rats in groups C, *E*, and ZnPP + *E* were bluntly separated from the right ureter, and the ureter was double-ligated with 4–0 silk suture near the lower pole of the right kidney, and then, the abdominal cavity was closed layer by layer. After 24 hours, rats in groups S and C were given normal saline (40 mg/kg/d) by gastric perfusion every day; rats in group *E* and group ZnPP + *E* were given ursolic acid preparation (40 mg/kg/d) by gastric perfusion every day, once a day, for 7 days. Among the rats in group ZnPP + *E*, after the right ureteral obstruction and before gastric infusion of ursolic acid preparations, intraperitoneal injection of zinc protoporphyrinn” is grammatically unclear. Please rephrase the part for clarity.” a specific blocker of Nrf2 signaling pathway (ZnPP, 45 umol/kg, dissolved in DMSO solution 0.5 ml). On the 8th day after operation, the right kidney tissue of the rat was taken for pathological examination.

### 2.3. Rat Kidney Function and Detection of MDA and SOD Content in Kidney Tissue

On the 8th day after the operation, the rats were anesthetized in the morning, and 2 ml of blood was taken from the abdominal aorta. After standing for 20 minutes, the rats were centrifuged at 4000 r/min for 15 minutes. The upper serum was collected and placed in an automatic biochemical analyzer to measure BUN and Cr levels (XR210, Shenzhen Medical Equipment Co., Ltd.). The right kidney of the rat was taken and normal saline was added to it to make the tissue homogenate. The instructions of the reagents were strictly followed; the thiobarbituric acid method was used to determine the content of kidney tissue MDA (BC0020, Solarbio) and the pyrogallol method to determine the kidney tissue SOD (S0103-1, Beyotime) activities.

### 2.4. Histopathological Examination of Rat Kidney

The right kidney tissue of the rat was taken and placed in 10% formalin solution for fixation. HE staining and Masson staining were routinely performed after alcohol gradient dehydration, xylene transparency, embedding, sectioning, and gradient deparaffinization. The operation process was carried out in strict accordance with the reagent instructions. The kidney tissue damage and fibrosis were observed under a light microscope (CX41, OLYMPUS), and Image-Pro Plus software was used to semi-quantitatively analyze the degree of renal interstitial fibrosis in rats. The evaluation method is as follows: each slice was randomly selected from 5 fields under a 200x optical microscope, and the percentage of the positive staining area in the entire field of view was calculated, and the average value was taken as the positive staining area of the specimen. All measurements were performed under the same optical conditions.

### 2.5. Immunohistochemical Detection of Rat Kidney Tissue

The EnVision two-step method was used for immunohistochemical staining; the instructions (Keap1 (10503-2-AP, Proteintech), Nrf2 (AF0639, Affinity), HO-1 (AF5393, Affinity), TGF-*β*1 (ABP52607, AmyJet Scientific), 8-oxo-dG (AF6980, Affinity)) were strictly followed. All the above detection indicators used PBS buffer instead of the primary antibody as a negative control. The above indicators were all positively expressed in the cytoplasm of renal tubular epithelial cells. Image-Pro Plus software was used to measure A values and perform protein semi-quantitative analysis.

### 2.6. TUNEL Method to Detect the Level of Apoptosis

The right kidney tissue of the rat was stained with TUNEL according to the operating instructions of the TUNEL detection kit (C1088, Biyuntian), and the operation process was carried out according to our previous study [8]. The labeling solution is used as a negative control, and deoxyribonuclease 1 (DNase1) is used as a positive control. The percentage of apoptotic cells in the total number of renal tubular epithelial cells was used as the apoptotic index (AI) of renal tubular epithelial cells.

### 2.7. RT-PCR

Use RT-PCR to evaluate the mRNA content of Keap1, Nrf2, HO-1, TGF-*β*1, 8-oxo-dG gene in kidney tissue, and the mRNA content of apoptosis protein caspase-3 and caspase-8 genes. The TRIzol method (CW0580S, CWBIO) was used to extract total RNA from rat kidney tissue, and cDNA was synthesized according to the instructions of the reverse transcription kit (CW0957M, CWBIO), and the target gene was amplified using cDNA as a template. The primer synthesis was completed by China Bioengineering (Shanghai) Co., Ltd., and the list of primers is shown in Table 1.

### 2.8. Western Blotting

The kidney tissue was thoroughly ground and added to the lysis solution, and centrifuged for 15 minutes in a 12000 r/min high-speed centrifuge. The BCA (CW0014S, Kangwei Century) method was used to determine the protein concentration and further determine the sample volume. Denaturation was performed at 90°C for 5 min and electrophoresis was carried out on 10% gel; afterwards, it was transferred to the PVDF membrane for blocking overnight. Then, the target antibody was added and incubated at room temperature for 3 hours; after washing the membrane with TBST, goat anti-rabbit IgG secondary antibody (1 : 2000) was added and incubated at room temperature for 2 hours; the membrane was washed with TBST and ECL was developed. ImageJ software was used to determine the ratio of the target band to the gray value of the internal reference *β*-actin, which reflects the protein expression level.

### 2.9. Data Analysis

The experimental data were statistically analyzed by SPSS 20.0 software, and the measurement data were expressed as mean ± standard deviation (mean ± SD), and the comparison between multiple groups was performed by the single-factor analysis of variance. The independent sample *t*-test was used for comparison between the two groups, and *P* < 0.05 considered the difference to be statistically significant.

## 3. Results

### 3.1. Ursolic Acid Protects Renal Function after Unilateral Ureteral Obstruction and Weakens Pathological Damage of Kidney Tissue

**Figure 1:**
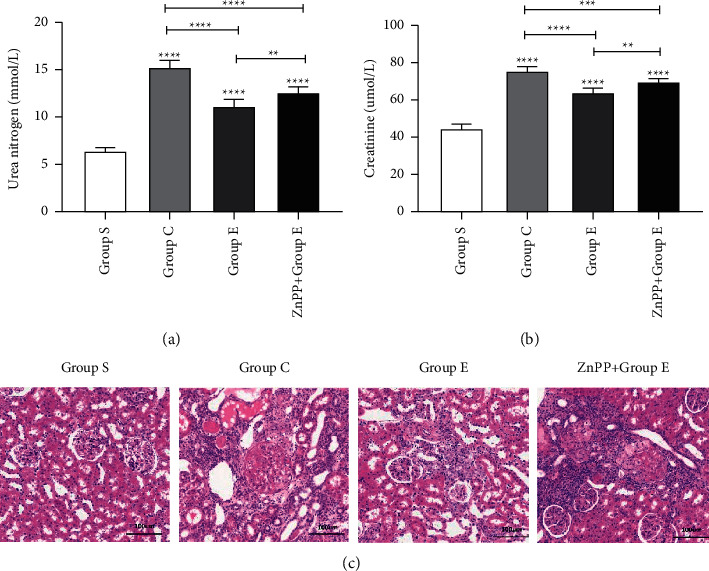
Ursolic acid relieves renal function damage and renal tissue pathological damage after unilateral ureteral obstruction in rats. (a) The changes in blood urea nitrogen levels of rats in each group on the 8th day after surgery (mean ± standard deviation, *n* = 6); (b) the changes in blood creatinine levels of rats in each group on the 8th day after surgery (mean ± standard deviation, *n* = 6); (c) special staining of rat kidney tissue with hematoxylin-eosin (HE) staining solution. On the 8th day after operation, after HE staining, the pathological damage of rat kidney tissue; ^*∗*^means *P* < 0.05; ^*∗∗*^means *P* < 0.01; ^*∗∗∗*^means *P* < 0.001; ^*∗∗∗∗*^means *P* < 0.0001.

Compared with group S, rats in groups C and *E* had significantly higher BUN and Cr on the 8th day after surgery, but the levels of BUN and Cr in group *E* were significantly lower than those in group C (Figures 1(a) and 1(b)). Prove that ursolic acid has a protective effect on renal function damage after unilateral ureteral obstruction. Through HE staining, we found that the morphology and structure of the kidney of group S rats were basically normal, and there was no obvious pathological damage. Mesangial cells in the glomeruli of rats in group C proliferate, the cells are tightly arranged, the glomerular cyst cavity disappears, and the basement membrane is thickened. Part of the renal tubular epithelial cells are edema, the cells are swollen and the cytoplasm is lightly stained, and the protein mucus in the lumen oozes out, and protein casts can be seen; the proliferation of collagen fibers with diffuse infiltration of inflammatory cells can be seen in the interstitium of renal tubules. Part of the glomeruli of rats in group E was slightly atrophy, the number of capillaries in the bulb was reduced, and the basement membrane was locally thickened; some renal tubular epithelial cells are slightly edema, the cells are swollen, and the cytoplasm is lightly stained; mild deposition of collagen fibers and proliferation of fibroblasts can be seen in the interstitium of some renal tubules (Figure 1(c)). It proves that ursolic acid can reduce the degree of pathological damage of kidney tissue after unilateral ureteral obstruction.

### 3.2. Ursolic Acid Reduces the Level of Renal Interstitial Fibrosis after Unilateral Ureteral Obstruction

**Figure 2:**
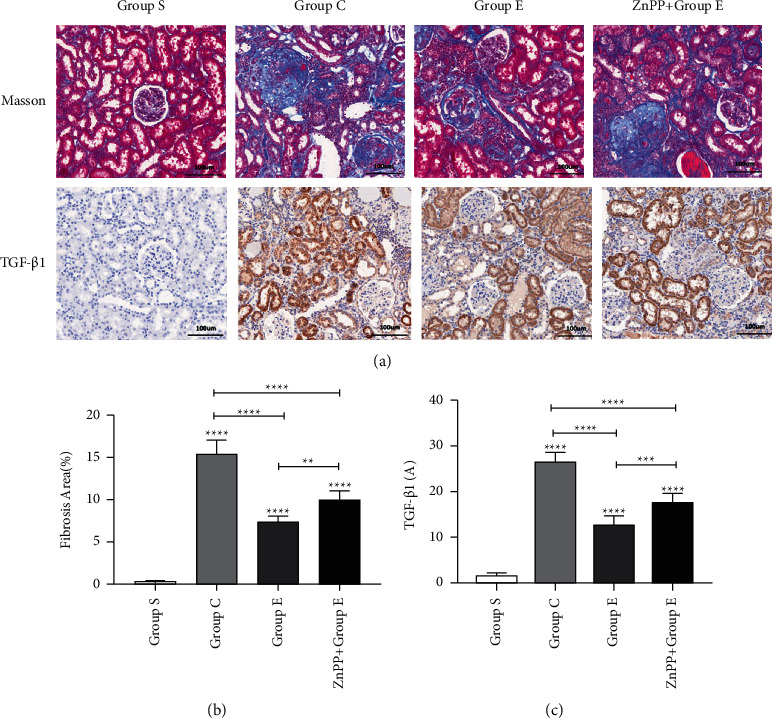
Ursolic acid reduces the degree of renal interstitial fibrosis after unilateral ureteral obstruction in rats. (a) The comparison of the degree of renal interstitial fibrosis in the kidney tissues of different groups of rats on the 8th day after Masson staining, and the expression of transforming growth factor-*β*1 (TGF-*β*1) in the kidney tissues of different groups of rats after immunohistochemical staining condition; (b) a statistical graph representing the percentage of positive areas of renal interstitial fibrosis in rats in different groups; (c) a statistical graph of TGF-*β*1 immunohistochemical absorbance (A) in the kidney tissues of rats in different groups; ^*∗*^means *P* < 0.05; ^*∗∗*^means *P* < 0.01; ^*∗∗∗*^ means *P* < 0.001; ^*∗∗∗∗*^means *P* < 0.0001.

**Figure 3:**
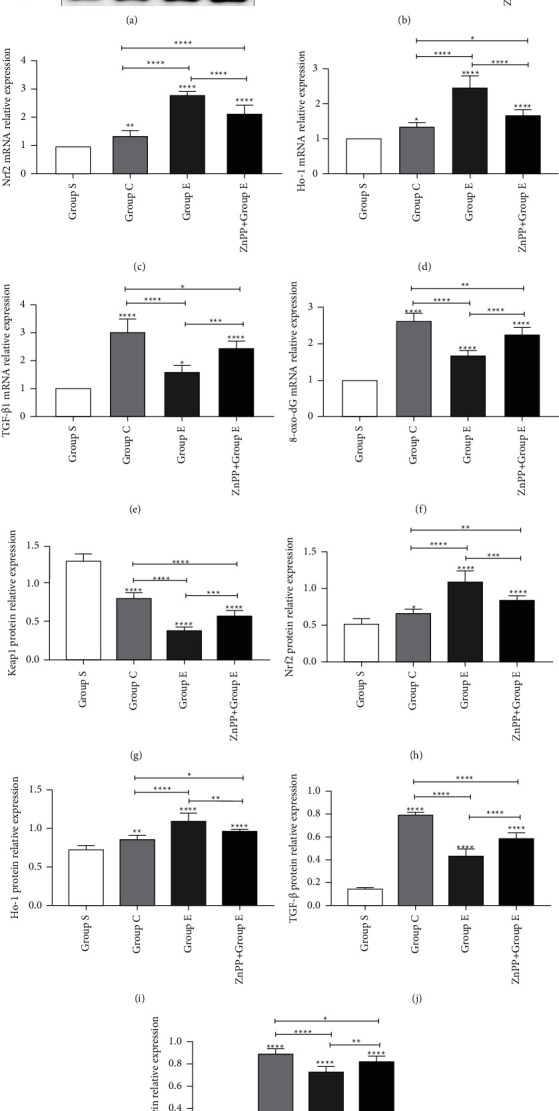
Ursolic acid activates the Nrf2 signaling pathway after unilateral ureteral obstruction in rats. (a) represents Western blots of related protein expression in rat kidney tissues of different groups; (b), (c), (d), (e), and (f) represent the expression of Keap1, Nrf2, HO-1, TGF-*β*1, and 8-oxo-dG mRNA in the kidney tissues of rats in different groups, respectively; (g), (h), (i), (j), and (k) represent the expression of Keap1, Nrf2, HO-1, TGF-*β*1, and 8-oxo-dG protein content, respectively, in the kidney tissue of rats in different groups; ^*∗*^means *P* < 0.05; ^*∗∗*^means *P* < 0.01; ^*∗∗∗*^means *P* < 0.001; ^*∗∗∗∗*^means *P* < 0.0001.

We found through Masson staining (Figures 2(a) and 2(b)) that there was no obvious fibrosis in the renal interstitium of rats in group S. The level of renal interstitial fibrosis in groups C and *E* rats was significantly higher than that in group S, but the level of renal interstitial fibrosis in group *E* was lower than that in group C. Prove that ursolic acid can attenuate the level of renal interstitial fibrosis after unilateral ureteral obstruction. Transforming growth factor-*β*1 (TGF-*β*1) is a recognized fibrogenic factor, which can induce renal interstitial fibrosis through a variety of signaling pathways. We detected TGF-*β*1 by immunohistochemistry (Figures 2(a) and 2(c)), RT-PCR (Figure 3(e)), and Western blotting (Figures 3(a) and 3(j)). Compared with group S, the expression of TGF-*β*1 mRNA and protein in the kidney tissue of rats in groups C and E increased significantly, but the level of TGF-*β*1 mRNA and protein in the kidney tissue of rats in group *E* was lower than those in group C. Once again, it was proved that ursolic acid can reduce the level of renal interstitial fibrosis after unilateral ureteral obstruction.

### 3.3. Ursolic Acid Reduces the Apoptosis Level of Renal Tubular Epithelial Cells after Unilateral Ureteral Obstruction

**Figure 4:**
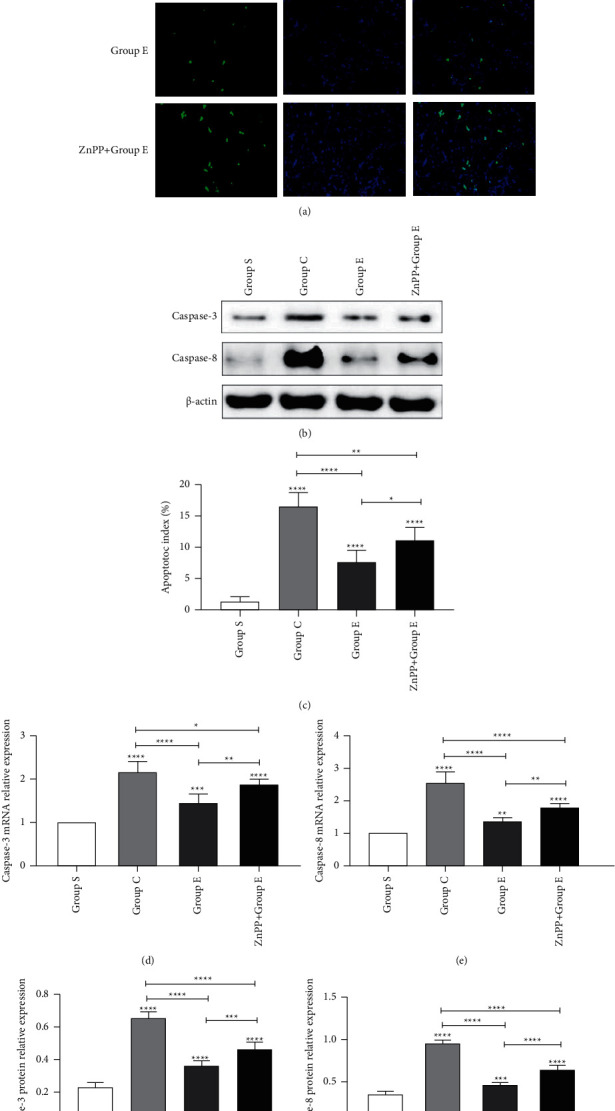
Ursolic acid reduces the apoptosis of renal tubular epithelial cells after unilateral ureteral obstruction in rats TUNEL staining is used to mark apoptotic cells; DAPI staining is used to mark normal cell nuclei; MERGE represents the combination of the two; observed under a fluorescence microscope, normal cell nuclei are blue, and apoptotic cell nuclei are green; (a) the apoptosis of renal tubular epithelial cells on the 8th day after operation in each group of rats; (b) the Western blot of caspase-3 and caspase-8 apoptotic proteins in each group of rats; (c) the statistical graph of the apoptosis index of renal tubular epithelial cells in each group (the percentage of the number of apoptotic cells in the total number of tubular epithelial cells is used as the apoptotic index (AI)) (mean ± standard deviation), *n* = 6); (d) the statistical graph of the apoptotic protein caspase-3 mRNA in each group; (e) the statistical graph of the apoptotic protein caspase-8 mRNA in each group; (f) the statistical graph of the expression level of the apoptotic protein Caspase-3; (g) the statistical graph of the expression level of the apoptotic protein caspase-8; ^*∗*^means *P* < 0.05; ^*∗∗*^means *P* < 0.01; ^*∗∗∗*^means *P* < 0.001; ^*∗∗∗∗*^means *P* < 0.0001.

Through TUNEL staining, we found that compared with group S, the apoptosis of renal tubular epithelial cells of rats in groups C and *E* increased significantly, but the apoptosis level of renal tubular epithelial cells of rats in group *E* was significantly lower than that in group C (Figures 4(a) and 4(c)). It is proved that ursolic acid can reduce the apoptosis level of renal tubular epithelial cells after unilateral ureteral obstruction. At the same time, we passed RT-PCR (Figures 4(d) and 4(e)) and Western blotting (Figures 4(b), 4(f), and 4(g)) to detect the apoptotic proteins caspase-3 and caspase-8. Compared with group S, the mRNA and protein levels of the apoptotic proteins caspase-3 and caspase-8 in C and *E* groups were significantly increased, but group *E* was significantly lower than that of group C. Once again, it was proved that ursolic acid can reduce the apoptosis level of renal tubular epithelial cells after unilateral ureteral obstruction.

### 3.4. Ursolic Acid Reduces the Oxidative Stress Level of Kidney Tissue after Unilateral Ureteral Obstruction

**Figure 5:**
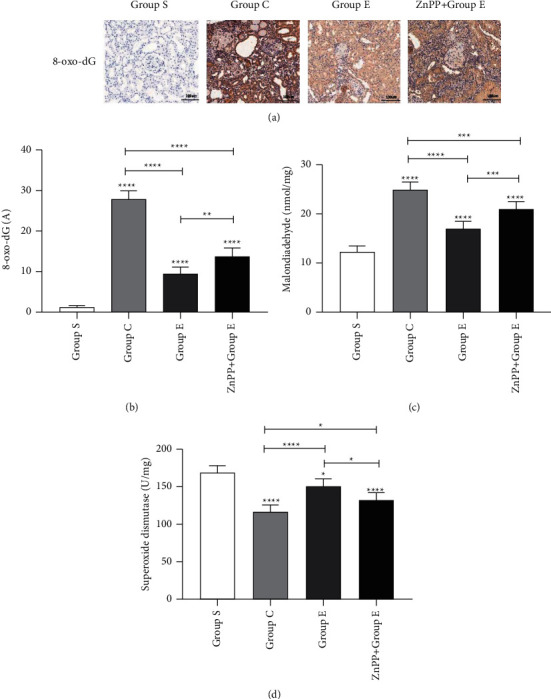
Ursolic acid reduces the level of oxidative stress in kidney tissue after unilateral ureteral obstruction in rats. (a) 8-oxo-dG immunohistochemical staining of oxidative stress damage marker in kidney tissue; (b) the statistical graphs of 8-oxo-dG immunohistochemical absorbance (A) values in rat kidney tissues of different groups; (c) the measurement of MDA content in kidney tissue by the thiobarbituric acid method (mean ± standard deviation, *n* = 6); (d) the SOD content in kidney tissue measured by the pyrogallol method (mean ± standard deviation, *n* = 6); ^*∗*^means *P* < 0.05; ^*∗∗*^means *P* < 0.01; ^*∗∗∗*^means *P* < 0.001; ^*∗∗∗∗*^means *P* < 0.0001.

8-Hydroxy-2-deoxyguanosine (8-oxo-dG) is widely used as a marker of oxidative stress damage in the body. In this study, we found through immunohistochemistry (Figures 5(a) and 5(b)), RT-PCR (Figure 3(f)), and Western blotting (Figures 3(a) and 3(k)) that compared with group S, the expression of 8-oxo-dG mRNA and protein in groups C and group E increased significantly. However, the expression in group E was significantly lower than that in group C, which proved that ursolic acid can effectively reduce oxidative stress damage in kidney tissue after unilateral ureteral obstruction in rats. Malondialdehyde (MDA) and superoxide dismutase (SOD) are often used together as markers to assess the level of oxidative stress in the body. In this study, compared with group S, the MDA content in group C and group E was significantly increased, but group E was significantly lower than group C (Figure 5(c)). Compared with group S, the SOD content in C and *E* groups was significantly reduced, but group *E* was significantly higher than group C (Figure 5(d)). It proves once again that ursolic acid can reduce the oxidative stress level of kidney tissue after unilateral ureteral obstruction in rats.

### 3.5. Ursolic Acid Plays a Protective Role by Activating the Nrf2/HO-1 Signaling Pathway

**Figure 6:**
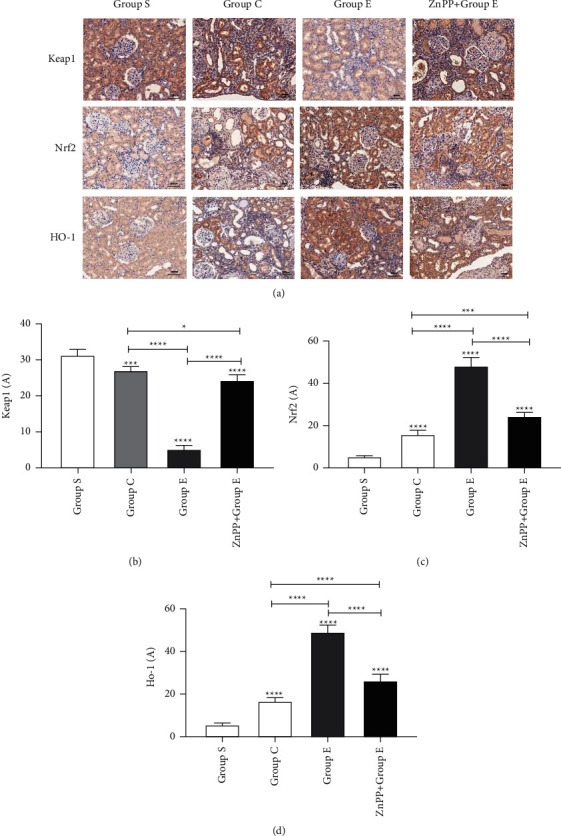
The immunohistochemical expression of ursolic acid in renal tissues of rats after unilateral ureteral obstruction. (a) The immunohistochemical expression map of related proteins in the kidney tissues of rats in different groups; (b), (c), (d) the statistical graphs of Keap1, Nrf2, and HO-1 immunohistochemical absorbance (a) values in the kidney tissues of rats in different groups, respectively; ^*∗*^means *P* < 0.05; ^*∗∗*^means *P* < 0.01; ^*∗∗∗*^means *P* < 0.001; ^*∗∗∗∗*^means *P* < 0.0001.

The Nrf2/HO-1 signaling pathway is the most common antioxidative stress pathway in tissues. Under normal circumstances, Nrf2 combines with its upstream inhibitor Keap1 and is widely distributed in tissues in an inactive form. When the body is stimulated by the external environment, the conformation of Keap1 changes, releases Nrf2, and stimulates the production of the downstream antioxidant substance HO-1, thereby playing a role in resisting oxidative stress damage. In this study, we found through RT-PCR (Figures 3(b)–3(d)), Western blotting (Figure 3(a), Figures 3(g)–3(i)), and immunohistochemistry (Figure 6). Compared with group S, the expression of Keap1 mRNA and protein in the kidney tissues of the rats in C and E groups was significantly decreased, and the decrease in group *E* was the most obvious. Compared with group S, the expression of Nrf2 and HO-1 mRNA and protein in groups C and E increased significantly, and group *E* increased the most. It is proved that ursolic acid can reduce the expression of Keap1 mRNA and protein, increase the expression of Nrf2 and HO-1 mRNA and protein, and activate the Nrf2/HO-1 signaling pathway, thereby exerting its protective effect.

### 3.6. After ZnPP Blocks the Nrf2/HO-1 Signaling Pathway, the Protective Effect Is Reversed

In order to further verify whether ursolic acid exerts a protective effect on renal interstitial fibrosis after unilateral ureteral obstruction in rats by activating the Nrf2/HO-1 signaling pathway, in this study, we established group ZnPP + *E* by the intraperitoneal injection of zinc protoporphyrin (ZnPP, 45 umol/kg, dissolved in DMSO solution 0.5 ml) into the rat's intraperitoneal cavity, which is a specific blocker of the Nrf2/HO-1 signaling pathway. Through the detection of rat kidney function (Figure 1), the detection of oxidative stress damage markers in kidney tissue (Figure 4), HE staining of rat kidney tissue (Figure 1), Masson staining (Figure 2), TUNEL staining (Figure 4), immunohistochemical staining (Figure 6), RT-PCR (Figure 3), and Western blotting (Figure 3) for related proteins were found. Compared with group E, rats in group ZnPP + *E* increased renal function damage and pathological damage, increased renal interstitial fibrosis, increased renal tubular epithelial cell apoptosis, and increased oxidative stress damage. It is proved that after ZnPP blocks the Nrf2/HO-1 signaling pathway, the protective effect of ursolic acid on kidney injury and renal interstitial fibrosis after unilateral ureteral obstruction in rats is reversed. At the same time through RT-PCR, Western blotting and immunohistochemical analysis results found that when ZnPP was applied, compared with group *E*, Keap1 mRNA and protein expression in the kidney tissue of group ZnPP + *E* increased, and the Nrf2 and HO-1 mRNA and protein expression decreased. Prove that ZnPP blocks the expression level of Nrf2/HO-1 signaling pathway. In summary, the protective effect of ursolic acid on renal interstitial fibrosis after unilateral ureteral obstruction in rats is mainly achieved by activating the Nrf2/HO-1 signaling pathway.

## 4. Discussion

In this study, we proved for the first time that ursolic acid can prevent renal interstitial fibrosis after unilateral ureteral obstruction, and its mechanism of action may be achieved by activating the Nrf2/HO-1 signaling pathway. We found in this experiment that after unilateral ureteral obstruction in rats, renal function is significantly impaired, renal tissue pathological damage is aggravated, renal interstitial fibrosis is significantly increased, and the apoptosis level of renal tubular epithelial cells is increased. When the rats were infused with ursolic acid preparations, the abovementioned injury was obviously alleviated; it was proved that ursolic acid preparations could alleviate the level of renal interstitial fibrosis in rats.

Renal interstitial fibrosis is the common result of the development of a variety of kidney diseases to the end stage, in which excessive deposition of extracellular matrix (ECM) is a typical pathological change of renal interstitial fibrosis [1]. Among the many cytokines that regulate renal interstitial fibrosis, transforming growth factor-*β*1 is currently the most studied cytokine with the most clear effect [9]. It has been confirmed that the upregulation of TGF-*β*1 is closely related to the excessive deposition of ECM [10]. Myofibroblasts are the main cells producing ECM. Some scholars have proved that the activation of renal interstitial fibroblasts to become *α*-SMA-positive myofibroblasts is the main factor of renal interstitial fibrosis injury [11]. Among them, TGF-*β*1 is its primary stimulus and activates fibroblasts through a variety of signaling pathways, for example, oxidative stress pathway [12] and Smad3 signaling pathway [13]. In our research, it was found that after unilateral ureteral obstruction in rats, the expression levels of TGF-*β*1 mRNA and protein in the kidney tissue were significantly higher than those in the sham operation group, and the degree of renal interstitial fibrosis was significantly increased. Given ursolic acid preparations, the expression levels of TGF-*β*1 mRNA and protein decreased, and the degree of renal interstitial fibrosis decreased. Once again, it proved the close relationship between TGF-*β*1 and the degree of renal interstitial fibrosis, which is often used as an important indicator of the prognosis of kidney disease.

Current research suggests that during oxidative stress damage, the production of reactive oxygen species (ROS) is considered to be an important factor leading to renal interstitial fibrosis [14]. Under normal circumstances, the production and removal of ROS in the body are in a dynamic balance. When the body is stimulated by exogenous or endogenous factors, a large amount of ROS is produced. Excessive ROS promotes the expression of inflammatory factors. initiates inflammations, and promotes the expression of fibrosis-related factors. For example, TGF-*β*, connective tissue growth factor, and platelet-derived growth factor (PDGF) accelerate the process of tissue fibrosis [10]. Therefore, weakening the body's oxidative stress response and reducing the production of ROS are considered to be an important treatment for alleviating renal interstitial fibrosis [15].

Ursolic acid is a natural triterpene carboxylic acid compound with anti-inflammatory, antibacterial, and antitumor effects [16]. Some scholars have proven that ursolic acid can resist tissue oxidative stress and have a protective effect on tissue damage [17]. The Nrf2/HO-1 signaling pathway is an important antioxidative stress pathway in the body. Under normal circumstances, Nrf2 binds to Kelch-like epichlorohydrin-related protein-1 (Keap1) and is widely distributed in the cytoplasm in an inactive form [18]. When the body is stimulated by oxidative stress, the conformation of Keap1 changes, dissociating Nrf2 from its inhibitory protein Keap1, and the dissociated Nrf2 migrates to the nucleus to form a heterodimer with Maf protein, which activates the expression of downstream antioxidant genes, for example, heme oxidase-1 (HO-1), SOD, and catalase, thereby increasing the ability of cells to resist oxidative stress [18–20]. Among them, HO-1 is the most important, an important inducible enzyme in the body. On the one hand, by reducing the production of heme, and on the other hand, by increasing the production of antioxidants such as CO and bilirubin, they can jointly play the role of antioxidative stress and anti-inflammatory [21, 22]. In this study, we found that when rats were given oral ursolic acid preparations, the expression levels of Keap1 mRNA and protein in the kidney tissue decreased, and the expression levels of Nrf2 and HO-1 mRNA and protein increased. The content of antioxidant substance SOD increased, and the levels of oxidative stress damage markers MDA and 8-oxo-dG decreased, which proves that the effect of ursolic acid against renal interstitial fibrosis may be achieved by activating the Nrf2/HO-1 signaling pathway. In order to further verify the feasibility of this pathway, we found that after the intraperitoneal injection of ZnPP, a specific blocker of the Nrf2/HO-1 signaling pathway in rats, compared with rats in the ursolic acid treatment group, the protein expression level of Keap1 mRNA in the kidney tissue increased, the expression levels of Nrf2 and HO-1 mRNA and protein decreased, the content of SOD decreased, and the levels of MDA and 8-oxo-dG increased, proving that the Nrf2/HO-1 signal pathway is blocked. Compared with rats treated with ursolic acid, this group of rats has worsened renal function damage, increased renal pathological damage, increased renal interstitial fibrosis, increased apoptosis, and increased TGF-*β* 1mRNA and protein expression levels. It was proved that after blocking the Nrf2/HO-1 signaling pathway, the protective effect of ursolic acid on renal interstitial fibrosis was reversed.

In summary, ursolic acid, as an antioxidant substance, can reduce the body's oxidative stress level, increase antioxidant substances, and reduce the expression of fibrotic factor TGF-*β*1 by activating the Nrf2/HO-1 signaling pathway. Thereby, it has a protective effect on the renal interstitium after unilateral ureteral obstruction in rats. Provide new ideas for clinical prevention and treatment of renal interstitial fibrosis.

## Figures and Tables

**Table 1:** Primer list.

Genes	Forward prime (5′-3′)	Reverse prime (5′-3′)
**Keap1**	GCCTTACTGTAGGCTGACGA	CTCCAGCTGCTAGACGGTCAT
**Nrf2**	CCATTTACGGAGACCCACC	GCTGTACTGTATCCCCAGAAGAA
**HO-1**	CAGCATGTCCCAGGATTTGT	TCACCAGCTTAAAGCCTTCC
**TGF-*β*1**	GACCGCAACAACGCAATCTATGAC	TGCTCCACAGTTGACTTGAATCTCTG
**8-Oxo-dG**	AAGTGTTCAGGTGGCCGGAT	TGGACAGTCTGCAGTTGGTT
**Caspase-3**	ATGGACAACAACGAAACCTC	CCACTCCCAGTCATTCCTT
**Caspase-8**	GGCCCTTCCTCGCTTCATCTC	GGTCCTTGGGCCTTCCTGGT
***β*-Actin**	GACCCTGAAGTACCCCATTG	GGTCATCTTTTCACGGTTGG

## Data Availability

The dataset can be accessed upon request.
